# Genome-wide analysis of gustatory receptor genes and identification of the fructose gustatory receptor in *Arma chinensis*

**DOI:** 10.1016/j.heliyon.2024.e30795

**Published:** 2024-05-09

**Authors:** Zhen Wang, Dianyu Liu, Le Ma, Hongmei Cheng, Changjin Lin, Luyao Fu, Yu Chen, Xiaolin Dong, Chenxi Liu

**Affiliations:** a Sino-American Biological Control Laboratory, Institute of Plant Protection, Chinese Academy of Agricultural Sciences, No. 2 Yuanmingyuan West Road, Haidian District, Beijing, 100193, China; b College of Agriculture, Yangtze University, No. 1 Nanhuan Road, Jingzhou, 434025, Hubei, China

**Keywords:** Gustatory receptor, Fructose, *Arma chinensis*

## Abstract

Gustatory receptors (GRs) allow insects to sense tastes in their external environment. Gustatory perception is crucial for distinguishing between beneficial and harmful or toxic compounds, affecting survival. This study is the first to identify and classify the GR genes and investigate their expression in the predatory *Arma chinensis*. Thirteen GR genes (*ArmaGr1*–*ArmaGr13*) were identified and classified into four families via phylogenetic analysis. In the predacious developmental stages, *ArmaGr7* expression gradually increased from the 2^nd^ to 5^th^ instar stages and then to adults. However, *ArmaGr7* was also highly expressed in the non-predation 1^st^ instar nymph and egg stages. *ArmaGr7* expression was localized in the antennae, scalpella, forelegs, wings, head, and midgut of male and female adults, with wings displaying the highest expression. Furthermore, *ArmaGr7* expression was positively correlated with fructose solution intake; molecular docking results showed that fructose could effectively dock withArmaGr7. A protein structure comparison revealed that the ArmaGr7 structure was different from that of other GR43a-like proteins, which may be related to the gene splicing of the *A. chinensis* GR gene. These results elucidate the crucial role of *ArmaGr7* in fructose recognition by *A. chinensis* and provide a foundation for further studies on gustatory perception.

## 1 Introduction

Gustatory receptor (GR) genes play a central role in coordinating insect feeding behaviors [1,2]. Most animals, particularly insects, rely on their taste sensory system to identify and evaluate potential foods by distinguishing nutrients that are beneficial to feeding behavior, growth, and metabolism from harmful or toxic compounds that are detrimental to survival [3].

The first GR genes in insects were identified in the *Drosophila melanogaster* genome [4] and have been classified into four clades: sugar [[5], [6], [7], [8]], bitter [9,10], CO_2_ [11], and GR43a-like [12]. As genomic detection technology has become more advanced, an increasing number of insect GRs have been discovered, including those of *Aedes aegypti* [13], *Halyomorpha halys* [14], *Tribolium castaneum* [15], *Bombyx mori* [9], and *Helicoverpa armigera* [16]. They provide valuable resources for in-depth studies on insect gustatory systems. The number of GR genes in each insect varies. For example, *D. melanogaster* and *A. aegypti* have 68 and 95 GR genes, respectively. *H. armigera* has 197 Gr genes, which may be related to the ability of *H. armigera* to feed on a wide variety of plants, presumably broadening its sense of plant secondary metabolites [17]. The human louse (*Pediculus humanus*) has only six GR genes, which may be related to obligate parasitism in humans [18].

GR genes are often expressed in gustatory sensilla, including the antennae [19], proboscises [20], maxillary palps [21,22], labial palps [23,24], tarsi [25,26], wings [27], and ovipositors [28]. Although some GR genes are expressed in multiple tissues, the expression of others is limited to specific developmental stages, organs, and tissues. For example, *HarmGR195* was detected only in adult tarsi, *HarmGR35* was detected only in adult heads, and *HarmGR65* was detected in larval fat bodies and adult male abdomens [17].

GR43a functions as a narrowly tuned fructose receptor in taste neurons [12,29]. In *D. melanogaster*, GR43a is essential for sensing hemolymph fructose concentrations, promoting feeding in hungry flies, and inhibiting feeding in full flies [29]. HarmGR4, a GR43a ortholog in *H. armigera*, was expressed in *Xenopus* oocytes, which responded to d-fructose in a dose-dependent manner when electrical signals were measured with a two-electrode voltage clamp [16,30]. BmGR9 specifically responds to fructose in *B. mori* and is expressed in cells of the oral sensory organs, including the maxillary galea, maxillary palps, labrum, and labium, as well as in putative neurosecretory cells of the central nervous system [31,32]. Although excellent progress has been made in understanding the roles of insect GR43a-like family members in taste perception, most studies in this regard have focused on pests. Few studies have examined the molecular mechanisms underlying taste perception in the natural enemies of pests.

*Arma chinensis* is a predatory stinkbug that uses external digestion to kill its prey and plays an important role in the control of lepidopteran and coleopteran pests [[33], [34], [35], [36]]. However, to date, only one study on *A. chinensis* has mentioned a partial GR gene [37]. Whole-genome analysis has allowed us to systematically understand the characteristics of specific gene families, including member classification, gene expression, and molecular evolution [38]. In the present study, we identified 13 GRs in *A. chinensis* and analyzed their phylogenetic relationships to other insect GRs. Further domain composition analysis revealed that the GR gene motifs in various subfamilies were different. We also studied the expression profiles of ArmaGr7 in *A. chinensis* that had been treated with different fructose concentrations for different periods. Structural alignment of this particular GR showed that the 3-dimensional (3D) structures differed from those of other insects. In summary, our study of the GR gene family in *A. chinensis* lays a genomic foundation for a better understanding of innate insect predation.

## 2 Materials and methods

### 2.1 Experimental insects

We used *A. chinensis* individuals belonging to a laboratory population that has been maintained for about 70 generations. The insects were fed *Antheraea pernyi* pupae and reared under conditions of 26 ± 1 °C, 65 ± 5 % relative humidity, and a 16 h light:8 h dark photoperiod. Healthy male and female insects with no apparent malformations or nutritional deficiencies were used for the bioassays.

### 2.2 Identification of GR genes in *A. chinensis*

To identify the members of the GR gene family in *A. chinensis*, published proteins of *D. melanogaster* were downloaded from the National Center for Biotechnology Information (NCBI) database (Supplementary Table 1). Subsequently, the published proteins were used as query sequences for BLAST 2.11.0+ against a database constructed using the predicted protein sequences of *A. chinensis* (GenBank Accession No. PRJNA660864), with a cutoff E-value of <10^−5^. The start and end sites of each gene on the chromosome and the length of each chromosome were obtained from the genome database, and a position map of the gene on the chromosome was drawn using TBtools-II (Toolbox for Biologists, v1.108). Next, the protein domains of the sequences from the previous step were searched against the Pfam protein database using HMMER v2.41.2. Motif-based analysis of the protein sequences was performed using the MEME online service (https://meme-suite.org/). All putative protein sequences from *A. chinensis* were aligned using ClustalW with the default parameters. A phylogenetic tree was generated using the MEGAX64 maximum likelihood method, with 1000 bootstrap repetitions.

### 2.3 Sequence alignment and phylogenetic analysis

Representative GR protein sequences from *A. aegypti*, *H. halys*, *T. castaneum*, *D. melanogaster*, *B. mori*, *H. armigera*, and *P. humanus* were obtained from the NCBI database. For phylogenetic analysis, all putative protein sequences from *A. chinensis* and the GR gene sequences of the aforementioned seven insect species were aligned using ClustalW with the default parameters. A phylogenetic tree was generated using the MEGAX64 neighbor-joining method, with 1000 bootstrap repetitions.

### 2.4 Real-time quantitative PCR

Real-time quantitative PCR was used to validate the expression of fructose GRs in *A. chinensis* at different developmental stages, in different organs, and after being fed with different concentrations of fructose. Total RNA was extracted from all samples using the LanEasy Total RNA kit (LanY Science & Technology Co., Ltd., Beijing, China) according to the manufacturer's instructions. RNA quality was assessed using a Nano-300 Microspectrophotometer (Hangzhou Allsheng Instruments Co., Ltd., Hangzhou, China) and RNA integrity was confirmed by agarose gel electrophoresis. First-strand cDNA was synthesized using a Fast Quant cDNA kit with gDNA Eraser (Tiangen Biotech Co., Ltd., Beijing, China) according to the manufacturer's instructions for quantitative reverse transcription PCR (qRT-PCR). Primers to amplify transcripts were based on *A. chinensis* sequences and designed using the Primer 3 v0.4.0 software (http://fokker.wi.mit.edu/primer3/) (Supplementary Table 2). qRT-PCR was carried out using a Quant Studio 5 thermocycler (Applied Biosystems, Foster City, CA, USA) with a PCR reaction volume of 20 μl, containing 2 μl of cDNA, 10 μl of 2 × T5 Fast qPCR Mix (TSINGKE Biotech Co., Ltd., Beijing, China), 0.8 μl of forward primer, 0.8 μl of reverse primer, 0.4 μl of 50 × ROX Reference Dye I, and 6 μl of nuclease-free water. The PCR cycling conditions comprised a 95 °C step for 1 min, followed by 40 cycles at 95 °C for 10 s, 60 °C for 5 s, and 72 °C for 10 s. All reactions were run in triplicate, with four independent biological replicates, and the dissociation curve was monitored to control for the potential formation of primer dimers. The mRNA expression levels were normalized against *A. chinensis* GAPDH and calculated using the 2^−ΔΔCt^ method [39].

### 2.5 Three-dimensional structural analysis and molecular docking

The *D. melanogaster* GR43a protein structure was downloaded from the Protein Data Bank database (https://www.rcsb.org/). All 3D structures of other target genes were constructed using the online prediction software Phyre2.0 (http://www.sbg.bio.ic.ac.uk/phyre2/) for homology modeling, and the optimal templates obtained for the modeling targets were selected based on high confidence, high sequence identity, and query coverage. The 3D structure of fructose was obtained from PubChem (https://pubchem.ncbi.nlm.nih.gov/) and was energy-optimized before the docking study using the molecular mechanics (MM2) method in ChemOffice Suite 2019 Chem3D software. The AutoDock Vina algorithm was employed for molecular docking studies [40]. Binding poses were obtained via energetic evaluation using the AutoDock Tools Score, and the optimized binding pose was displayed using PyMOL Win v2.4.0). In addition, VMD v1.9.4a53 software was employed to calculate the root-mean-square deviations (RMSD) between structures.

### 2.6 Statistical analyses

Statistical analyses were performed using GraphPad Prism v8.0.1 (GraphPad Software, San. Diego, CA, USA) or SPSS (SPSS 24 software, Chicago, IL, USA). Relative expression levels were analyzed using one-way ANOVA (SPSS) with the Waller–Duncan post hoc comparison test. Data are presented as the mean ± standard error of the mean (SEM). Differences were considered statistically significant at P < 0.05. Graphs were constructed using GraphPad Prism, unless otherwise specified.

## 3 Results

### 3.1 GR genes in *A. chinensis*

**Fig. 1:**
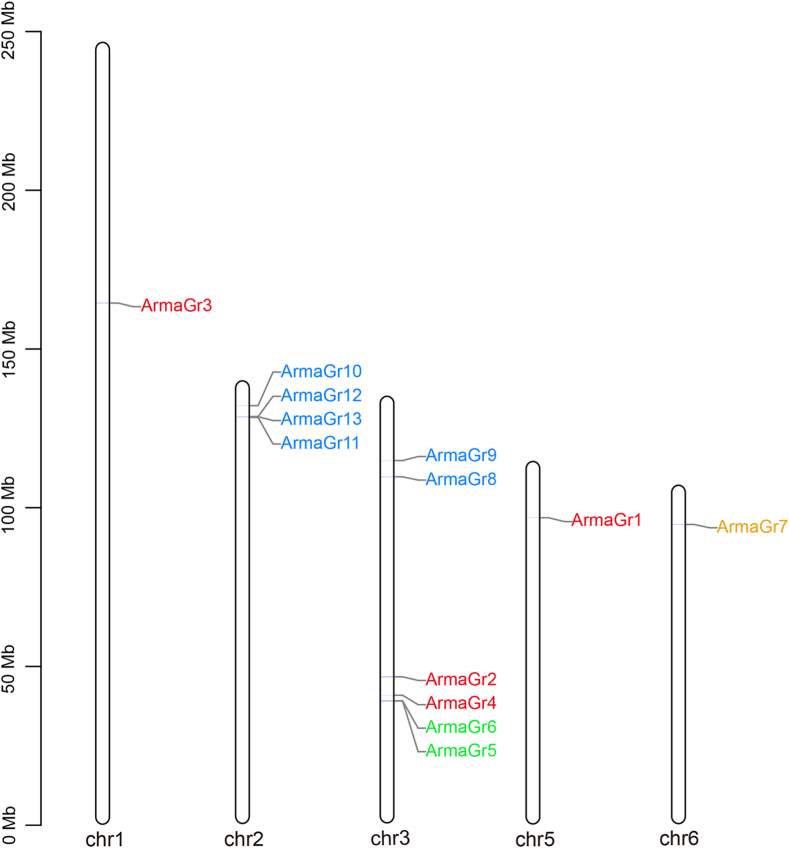
Genome-wide distribution of 13 putative gustatory receptor genes in *Arma chinensis*. The red gene name represents CO_2_ receptors, green denotes sugar receptors, yellow fructose receptors, and blue bitter receptors.

We identified 13 GR genes in the *A. chinensis* genome database and named them *ArmaGr1*–*13*. Among these, there were six bitter GRs (46 % of the total), four CO_2_ GRs (31 %), two sugar GRs (15 %), and one fructose GR. The loci of these 13 genes were mapped to five putative *A. chinensis* chromosomes using chromosomal location analysis (Fig. 1). Genes with the same function were scattered around different chromosomes instead of being linked, and the sizes of proteins they expressed were variable, ranging from 321 (ArmaGr4) to 721 (ArmaGr2) residues (Supplementary Table 3). For example, the CO_2_ GRs (*ArmaGr1*, *ArmaGr2*, *ArmaGr3*, and *ArmaGr4*) are located on three different chromosomes, while the bitter GRs (*ArmaGr10*, *ArmaGr11*, *ArmaGr12*, and *ArmaGr13*) are located in close proximity on the same chromosome.

**Fig. 2:**
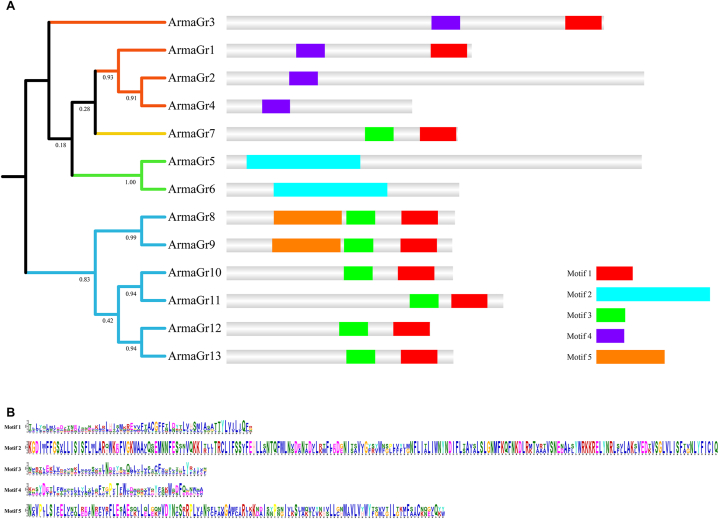
A: Phylogenetic relationship and protein motif analyses of the *A. chinensis* gustatory receptors. The unrooted phylogenetic tree was constructed using the MEGAX maximum likelihood method and a bootstrap test was performed with 1000 replicates. The colored shadow marks the different ArmaGr families. All motifs were identified via the MEME database using the complete amino acid sequences of ArmaGrs. Lengths of motifs for each ArmaGr protein are exhibited proportionally. B: Sequence logos of the conserved motifs of ArmaGr proteins in *A. chinensis*. The overall height of each column is proportional to the information content of all amino acids at that position; within each column, the conservation of each residue is visualized as the relative height of the symbols representing amino acids.

A phylogenetic tree was constructed based on the sequences of all 13 *A. chinensis* GR proteins using the maximum likelihood method with 1000 bootstrap replicates (Fig. 2). The results showed that all the CO_2_ GR proteins had motif 4, bitter and fructose GR proteins all had motifs 1 and 3, and sugar GR proteins all had motif 2. This indicates that different motifs may perform different functions. Interestingly, fructose and bitter GR proteins shared the same motifs.

### 3.2 Phylogenetic analysis of insect GR genes

**Fig. 3:**
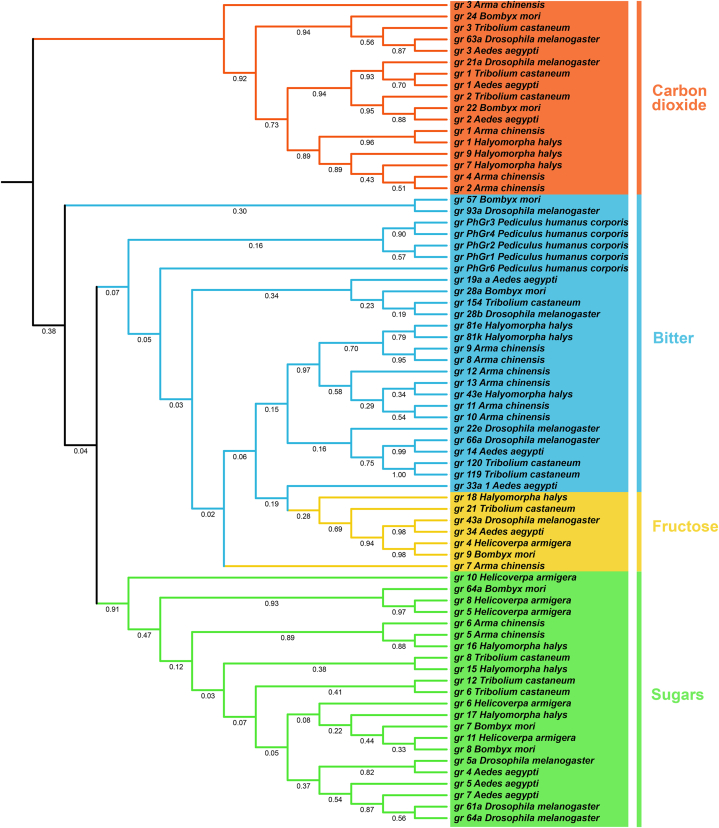
Phylogenetic analysis of *Aedes aegypti*, *Halyomorpha halys*, *Tribolium castaneum*, *Drosophila melanogaster*, *Bombyx mori*, *Helicoverpa armigera*, *Pediculus humanus*, and *A. chinensis*. The unrooted phylogenetic tree was constructed using the MEGAX neighbor-joining method and a bootstrap test was performed with 1000 replicates. The specific colors indicate different families.

The amino acid sequences of the 13 *A. chinensis* GRs were aligned with 59 GRs from seven other insect species using ClustalW, and a neighbor-joining phylogenetic tree containing GR sequences from all eight insects was constructed (Fig. 3). Overall, the insect GRs were divided into four groups: CO_2_, bitter, fructose, and sugars. Bitter GRs constituted the largest group (26 GRs). The 13 *A. chinensis* GRs were divided into the four groups as follows: *ArmaGr1–4* belonged to the CO_2_ group, both *ArmaGr5* and *ArmaGr6* belonged to the sugar group, the fructose group contained *ArmaGr7*, and the bitter group contained *ArmaGr8–13*.

### 3.3 Tissue, instar, and gender specific expression profiles of ArmaGr7

**Fig. 4:**
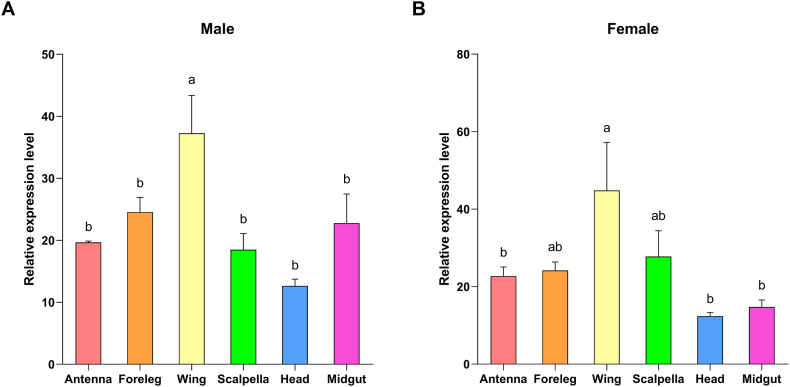
Quantitative real-time polymerase chain reaction (qPCR) analysis of expression profiles of ArmaGr7 genes in different tissues of *A. chinensis*. Different tissues are indicated with different colors, and the expression levels are shown on the *y*-axis. Data shown are mean relative expression levels ± SE. Data were analyzed using one-way analysis of variance (P < 0.05) with the Waller–Duncan post hoc test (n = 3). Different letters on error bars indicate significant differences.

We determined the expression levels of *ArmaGr7* in the antennae, scalpella, forelegs, wings, head (excluding the antennae and scalpella), and midgut of male and female *A. chinensis*. Thirty adult *A. chinensis* were selected for tissue sampling, and biological replicates were performed thrice for males and females. The results showed that the expression level of *ArmaGr7* was the highest in the wings but was lower in other tissues, with no significant difference between them (Fig. 4A and B).

**Fig. 5:**
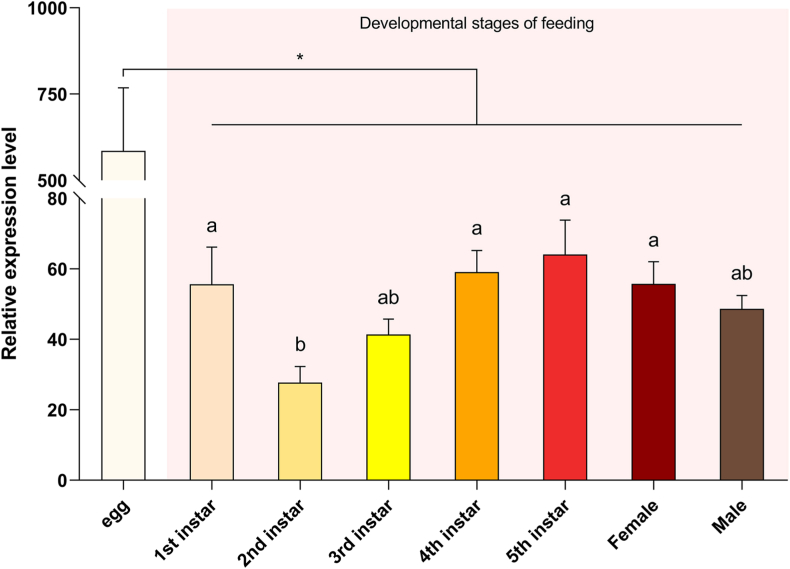
qPCR analysis of ArmaGr7 gene expression profiles in different instar nymph stages and adults of *A. chinensis*. The expression levels are shown on the y-axis. Data reflect the mean relative expression levels ± SE. Data were analyzed using one-way analysis of variance (P < 0.05) with the Waller–Duncan post hoc test (n = 3). Different letters on error bars indicate significant differences.

The expression levels of *ArmaGr7* were measured in eggs, nymphs, adult males, and adult females. Five egg masses and ten *A. chinensis* each were selected for nymphs, adult males, and adult females, and three biological replicates were used. The results showed that the expression levels of *ArmaGr7* were much lower in the 2^nd^ and 3^rd^ instar and reached the highest level in the 5^th^ instar (Fig. 5).

### 3.4 Response of ArmaGr7 to fructose variable treatments

**Fig. 6:**
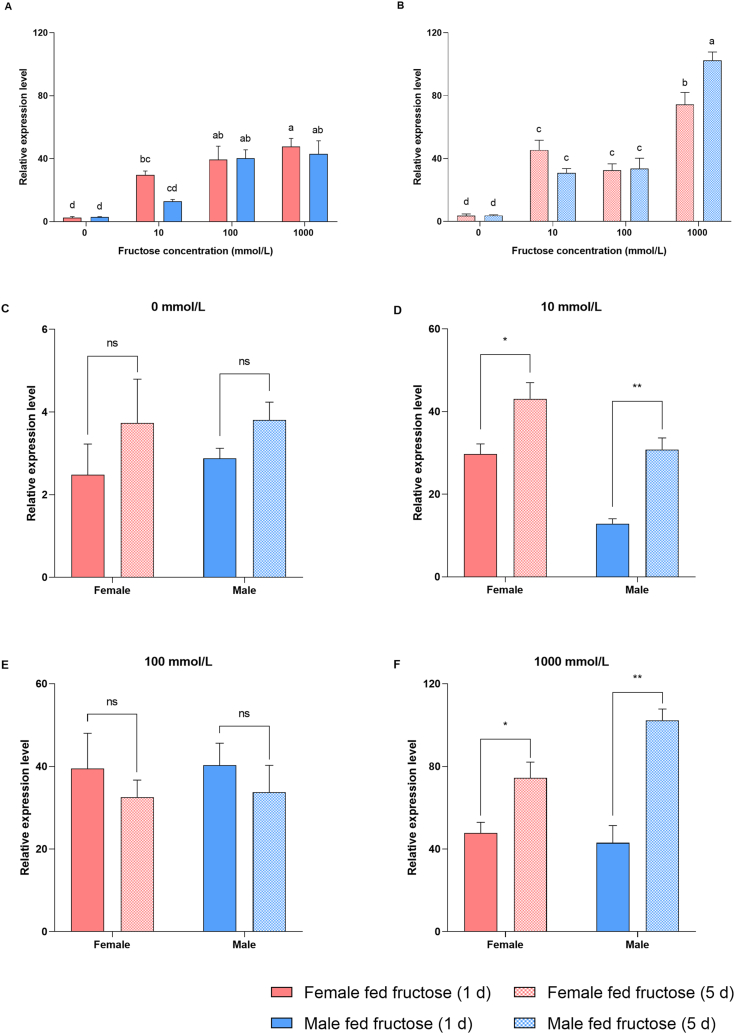
The ArmaGr7 expression level was measured in *A. chinensis* that were fed with 10, 100, or 1000 mmol/L fructose solution for 1 or 5 d. Data shown are the mean relative expression levels ± SE. A, B: Data were analyzed using one-way analysis of variance (P < 0.05 with the Waller–Duncan post hoc test (n = 3). Different letters on error bars indicate significant difference. C–F: Data were analyzed using two-tailed Student's t tests (n = 3, mean ± SE; ns: P > 0.05, *P < 0.05, **P < 0.01). ns: no significant difference found.

Male and female *A. chinensis* were fed 10, 100, or 1000 mmol/L fructose solutions for one or five days; in a control group, male and female adults of the same age were fed water. Each treatment was performed for three biological replicates of *A. chinensis*, with each replicate containing 10 adults. The results showed that *ArmaGr7* expression was positively correlated with the concentration of fructose solution after its consumption for either 1 or 5 d (Fig. 6 A - F).

The expression of *ArmaGr7* in *A. chinensis* fed with fructose solution was significantly higher than that in insects in the control group. After 1 day of treatment, *ArmaGr7* expression increased with the increase in fructose concentration, but there was no significant difference in *ArmaGr7* expression between the 100 mmol/L and 1000 mmol/L groups, indicating that *ArmaGr7* expression would not continue to increase when fructose concentration was higher than 100 mmol/L (Fig. 6A). When feeding time was extended to 5 days, the expression of *ArmaGr7* in the 1000 mmol/l fructose solution group was significantly higher than that in the 100 mmol/l group. This suggests that *ArmaGr7* accumulates over time and that this process is facilitated by higher fructose solution concentrations (Fig. 6B).

### 3.5 3D structure prediction and conservative analysis of ArmaGr7

**Fig. 7:**
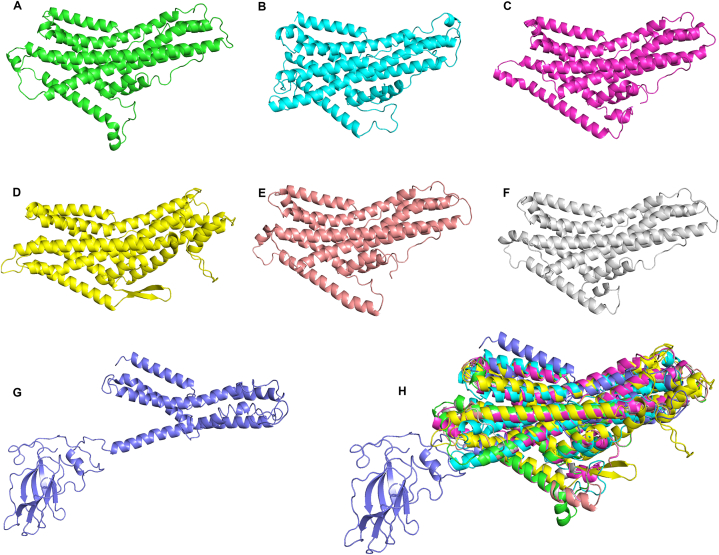
The 3D structures of fructose gustatory receptor domains are conserved in different insects, but ArmaGr7 differed from other GR43a-like proteins. A–G: 3D structures of fructose gustatory receptor domains from *A. aegypti*, *H. halys*, *T. castaneum*, *D. melanogaster*, *B. mori*, *H. armigera*, and *A. chinensis.* H: Structural alignments of fructose gustatory receptor domains from different insects; the colors correspond to A–G, respectively.

**Fig. 8:**
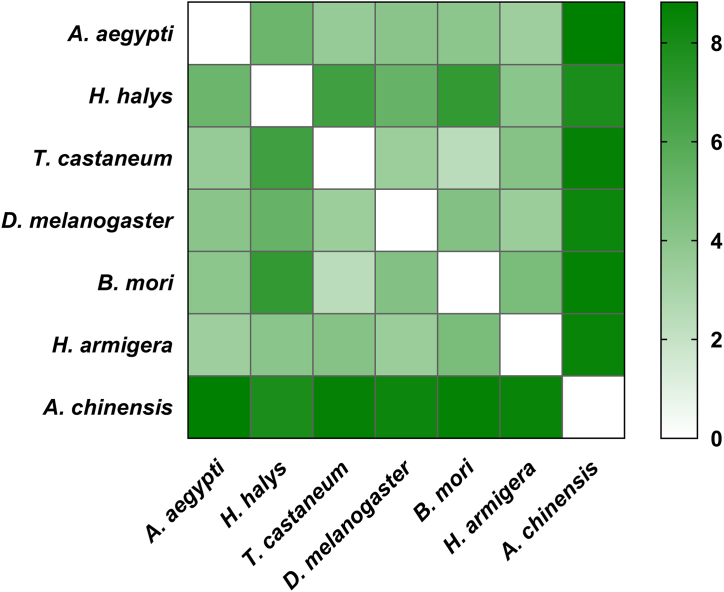
Root-mean-square deviation (RMSD) matrix of fructose gustatory receptor domains from different insects. The color gradient of the left color block represents the relative similarity of gr43a-like receptors between the corresponding species, ranging from strong to weak.

The primary and tertiary structures of various protein domains that play important roles in organisms are evolutionarily conserved. To investigate the structural characteristics of the GR domains of different insects, we obtained the 3D structure of the *D. melanogaster* GR43a domains and predicted the GR43a-like 3D structures of *A. aegypti, H. halys, T. castaneum, B. mori,* and *H. armigera*. The predictions depicted 9–10 α-helixes with components arranged in similar patterns (Fig. 7A–F). A comparison of the *D. melanogaster* GR43a structure with the fructose GR domains of *A. chinensis* revealed that *D. melanogaster* contained one β-sheet and ArmaGr7 contained only 6 α-helixes but 7 β-sheets (Fig. 7C and G). The 3D structural alignments showed that fructose GR domains from different species (except for *A. chinensis*) were similar in spatial conformation (Fig. 7H) and that the RMSD ranged from 2.12 to 6.60, suggesting that fructose GR domains were structurally conserved during evolution (Fig. 8). The RMSD values of *A. chinensis* with other insects were in the range of 7.87–8.82, suggesting that there are structural variations between the ArmaGr7 of *A. chinensis* and other insects.

### 3.6 Binding of ArmaGr7 to fructose

**Fig. 9:**
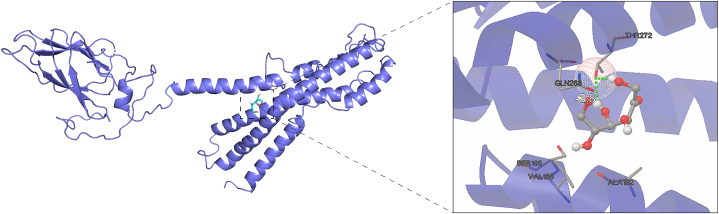
Fructose docked into the binding site of ArmaGr7. The receptor is represented as a cartoon, and the involved residues are shown in lines. The fructose molecule is presented in the stick mode, and the two hydrogen bonds are presented as green dashed lines.

Fructose docked successfully with ArmaGr7, which adopted a compact conformation to bind in the “pocket” of this protein. Fructose is surrounded by three hydrophilic residues (THR272, GLN268, and SER186) and two hydrophobic residues (VAL185 and ALA182) (Fig. 9). The hydroxide radical on the fructose I and IV carbon could form hydrogen bonds (distances of 1.8 Å and 2.0 Å) with the oxygen atom on THR272 (Fig. 9).

## 4 Discussion

This is the first report on GR genes of *A. chinensis*. Compared with the relatively large number of these genes in common pests, such as *H. halys* [14], *Spodoptera frugiperda* [[41], [42], [43]], and *H. armigera* [16], there are only 13 GR genes in *A. chinensis*, which represents a reduction of approximately 90 %. In comparison, *Rhodnius prolixus* is a predatory insect with 28 GR genes, and *Cimex lectularius*, a blood-sucking insect, possesses 24 GR genes [14]. The expansion and contraction of receptor family sizes across arthropods reveals a generally positive correlation with their widely disparate complexity of chemical ecology [[44], [45], [46]]. The number of GR genes may be related to insect feeding habits.

We conducted a systematic bioinformatics analysis of the GR genes in *A. chinensis* and classified them into subfamilies. Despite possessing less GR genes overall, *A. chinensis* had a relatively fraction of genes in the bitter GR subfamily compared to other insects [[13], [14], [15], [16]]. In addition, the proportion of the CO_2_ GR gene subfamily among all GRs was significantly increased in this species. This may be related to the ability of *A. chinensis* to locate its prey by sensing CO_2_ [47]. As in other insects, the proportion of fructose GR genes was the lowest in *A. chinensis*. Motif prediction showed that GR genes with different functions have different motifs. Interestingly, fructose GR and bitter GR proteins had the same motif and were on the same branch of the evolutionary tree. This suggests that the two types of GR may have a close evolutionary relationship [48].

In the gustatory system of *D. melanogaster*, the most prominent expression of GR43a is found in the legs [49], while that of GR43aGAL4 is much lower in the labial palp than in any other organ [29]. In addition, GR43a is expressed in defined sets of neurons in the proventricular ganglion, brain, and uterus [29]. In *Plutella xylostella*, PxylGR43a-1 is predominantly expressed in the antennae of male and female moths, but weakly expressed in male and female heads and male legs [50]. Electrophysiological analysis of *A. aegypti* tissues provided evidence that the tarsi and wings could sense chemicals in a gaseous form [51]. In the present study, *ArmaGr7* was expressed in the antennae, scalpella, forelegs, wings, head, and midgut, and the expression level of *ArmaGr7* was significantly higher in the wings than in other tissues. This suggests that *A. chinensis* wings can be used to detect fructose. The higher expression level of *ArmaGr7* in the 1^st^ instar can be related to the feeding behavior of most Pentatomidae neonates, which consume carbohydrates on the egg surface (Fig. 5). However, we found that *ArmaGr7* expression was approximately 10 times higher in the egg stage than in any other developmental stages, suggesting that *ArmaGr7* fulfils other potential roles.

In a study investigating the predation functional response of *Cryptolaemus montrouzieri* against *Dysmicoccus neobrevipes*, the results showed that predation ability increased with nymph instar progression [52]. Our RT-qPCR assay findings indicated that *ArmaGr7* expression increased with the *A. chinensis* nymph instar progression, and the expression levels peaked in the 5^th^ instar nymphs. An increasing predatory ability in *A. chinensis* nymph might be accompanied with an increase in *ArmaGr7* expression.

After feeding *A. chinensis* with 10 or 1000 mmol/L fructose solution, the expression level of *ArmaGr7* was significantly higher after 5 d than after only 1 d; however, there was no significant difference in *ArmaGr7* expression after either 1 or 5 d when feeding insects with 100 mmol/L of the fructose solution. Therefore, *ArmaGr7* expression may be related to fructose accumulation. When fructose accumulation in *A. chinensis* exceeds a certain threshold, the expression level of *ArmaGr7* increases significantly. In addition, after 1 d of feeding *A. chinensis* with 100 or 1000 mmol/L fructose solution, there was no significant difference in the expression levels of *ArmaGr7*; however, after 5 d, *ArmaGr7* expression had increased significantly in those individuals fed with a 1000 mmol/L fructose solution. This may be due to the rapid accumulation of fructose at high concentrations.

The structural alignment of fructose GR domains from different insects revealed that they are highly conserved and possess similar structural components and spatial arrangements. The molecular docking results also showed that ArmaGr7 could effectively bond with fructose molecules and form hydrogen bonds. This provided additional evidence that ArmaGr7 recognizes fructose. However, *A. chinensis* has more β-sheets in its ArmaGr7 protein structure compared to other insects. This may be due to sequence splicing of the GR gene in *A. chinensis*. The function of the extra β-sheets requires further investigation.

In conclusion, we conducted an in-depth study of the fructose GRs in *A. chinensis*. We investigated the main gene involved in the perception of fructose and the changes in *ArmaGr7* expression when these insects were fed different concentrations of fructose solution. This study provides data for future studies on *A. chinensis*, including research on the effects of artificial diets, and it lays the groundwork for the study of GR genes in other insects.

## Ethics approval

This study required no ethical approval.

## Data availability statement

Data will be made available on request.

## CRediT authorship contribution statement

**Zhen Wang:** Writing – original draft, Validation, Methodology, Investigation, Formal analysis, Data curation. **Dianyu Liu:** Validation, Data curation. **Le Ma:** Investigation, Data curation. **Hongmei Cheng:** Investigation, Data curation. **Changjin Lin:** Investigation, Data curation. **Luyao Fu:** Investigation, Data curation. **Yu Chen:** Investigation, Data curation. **Xiaolin Dong:** Methodology, Investigation, Data curation, Conceptualization. **Chenxi Liu:** Writing – review & editing, Writing – original draft, Validation, Methodology, Investigation, Funding acquisition, Data curation, Conceptualization.

## Declaration of competing interest

The authors declare that they have no known competing financial interests or personal relationships that could have appeared to influence the work reported in this paper.

## References

[1] van der Goes van Naters W., Carlson J.R. (2006). Insects as chemosensors of humans and crops. Nature.

[2] Liman E.R., Zhang Y.V., Montell C. (2014). Peripheral coding of taste. Neuron.

[3] Scott K. (2005). Taste recognition: food for thought. Neuron.

[4] Clyne P.J., Warr C.G., Carlson J.R. (2000). Candidate taste receptors in *Drosophila*. Science.

[5] Jiao Y., Moon S.J., Montell C. (2007). A *Drosophila* gustatory receptor required for the responses to sucrose, glucose, and maltose identified by mRNA tagging. Proc. Natl. Acad. Sci. U. S. A..

[6] Dahanukar A., Lei Y.T., Kwon J.Y., Carlson J.R. (2007). Two *Gr* genes underlie sugar reception in *Drosophila*. Neuron.

[7] Jiao Y., Moon S.J., Wang X., Ren Q., Montell C. (2008). Gr64f is required in combination with other gustatory receptors for sugar detection in *Drosophila*. Curr. Biol..

[8] Slone J., Daniels J., Amrein H. (2007). Sugar receptors in *Drosophila*. Curr. Biol..

[9] Wanner K.W., Robertson H.M. (2008). The gustatory receptor family in the silkworm moth *Bombyx mori* is characterized by a large expansion of a single lineage of putative bitter receptors. Insect Mol. Biol..

[10] Lee Y., Moon S.J., Montell C. (2009). Multiple gustatory receptors required for the caffeine response in *Drosophila*. Proc. Natl. Acad. Sci. U. S. A..

[11] Jones W.D., Cayirlioglu P., Kadow I.G., Vosshall L.B. (2007). Two chemosensory receptors together mediate carbon dioxide detection in *Drosophila*. Nature.

[12] Sato K., Tanaka K., Touhara K. (2011). Sugar-regulated cation channel formed by an insect gustatory receptor. Proc. Natl. Acad. Sci. U. S. A..

[13] Erdelyan C.N., Mahood T.H., Bader T.S., Whyard S. (2012). Functional validation of the carbon dioxide receptor genes in *Aedes aegypti* mosquitoes using RNA interference. Insect Mol. Biol..

[14] Sparks M.E., Bansal R., Benoit J.B. (2020). Brown marmorated stink bug, *Halyomorpha halys* (Stål), genome: putative underpinnings of polyphagy, insecticide resistance potential and biology of a top worldwide pest. BMC Genom..

[15] Richards S., Gibbs R.A. (2008). The genome of the model beetle and pest *Tribolium castaneum*. Nature.

[16] Jiang X.J., Ning C., Guo H. (2015). A gustatory receptor tuned to ᴅ-fructose in antennal sensilla chaetica of *Helicoverpa armigera*. Insect Biochem. Mol. Biol..

[17] Xu W., Papanicolaou A., Zhang H.J., Anderson A. (2016). Expansion of a bitter taste receptor family in a polyphagous insect herbivore. Sci. Rep..

[18] Guo H., Cheng T., Chen Z. (2017). Expression map of a complete set of gustatory receptor genes in chemosensory organs of *Bombyx mori*. Insect Biochem. Mol. Biol..

[19] Yang K., Gong X.L., Li G.C., Huang L.Q., Ning C., Wang C.Z. (2020). A gustatory receptor tuned to the steroid plant hormone brassinolide in *Plutella xylostella* (Lepidoptera: plutellidae). Elife.

[20] Zhu J.Y., Xu Z.W., Zhang X.M., Liu N.Y. (2018). Genome-based identification and analysis of ionotropic receptors in *Spodoptera litura*. Naturwissenschaften.

[21] Roessingh P., Xu S., Menken S.B.J. (2007). Olfactory receptors on the maxillary palps of small ermine moth larvae: evolutionary history of benzaldehyde sensitivity. J. Comp. Physiol. A Neuroethol. Sens. Neural Behav. Physiol..

[22] Hall L.P., Graves F., Myrick A., Hoover K., Baker T.C. (2019). Labial and maxillary palp recordings of the Asian longhorned beetle, *Anoplophora glabripennis*, reveal olfactory and hygroreceptive capabilities. J. Insect Physiol..

[23] Guo M., Chen Q., Liu Y., Wang G., Han Z. (2018). Chemoreception of mouthparts: sensilla morphology and discovery of chemosensory genes in proboscis and labial palps of adult *Helicoverpa armigera* (Lepidoptera: noctuidae). Front. Physiol..

[24] Du L., Zhao X., Liang X., Gao X., Liu Y., Wang G. (2018). Identification of candidate chemosensory genes in *Mythimna separata* by transcriptomic analysis. BMC Genom..

[25] Sparks J.T., Vinyard B.T., Dickens J.C. (2013). Gustatory receptor expression in the labella and tarsi of *Aedes aegypti*. Insect Biochem. Mol. Biol..

[26] Seada M.A., Ignell R., Al Assiuty A.N., Anderson P. (2018). Functional characterization of the gustatory sensilla of tarsi of the female polyphagous moth *Spodoptera littoralis*. Front. Physiol..

[27] Yanagawa A., Couto A., Sandoz J.C. (2019). LPS perception through taste-induced reflex in *Drosophila melanogaster*. J. Insect Physiol..

[28] Seada M.A., Ghaninia M. (2016). Deep-tissue confocal imaging of the central projections of ovipositor sensory afferents in the Egyptian cotton leafworm, *Spodoptera littoralis*. Micron.

[29] Miyamoto T., Slone J., Song X., Amrein H. (2012). A fructose receptor functions as a nutrient sensor in the *Drosophila* brain. Cell.

[30] Zhang Y.F., van Loon J.J., Wang C.Z. (2010). Tarsal taste neuron activity and proboscis extension reflex in response to sugars and amino acids in *Helicoverpa armigera* (Hubner). J. Exp. Biol..

[31] Mang D., Shu M., Tanaka S. (2016). Expression of the fructose receptor BmGr9 and its involvement in the promotion of feeding, suggested by its co-expression with neuropeptide F1 in *Bombyx mori*. Insect Biochem. Mol. Biol..

[32] Xu W., Zhang H.J., Anderson A. (2012). A sugar gustatory receptor identified from the foregut of cotton bollworm *Helicoverpa armigera*. J. Chem. Ecol..

[33] Zou D., Coudron T.A., Liu C., Zhang L., Wang M., Chen H. (2013). Nutrigenomics in *Arma chinensis*: transcriptome analysis of *Arma chinensis* fed on artificial diet and Chinese oak silk moth *Antheraea pernyi* pupae. PLoS One.

[34] Zou D., Coudron T.A., Wu H. (2022). Differential proteomics analysis unraveled mechanisms of *Arma chinensis* responding to improved artificial diet. Insects.

[35] Zou D.Y., Coudron T.A., Wu H.H. (2015). Performance and cost comparisons for continuous rearing of *Arma chinensis* (Hemiptera: Pentatomidae: Asopinae) on a Zoophytogenous artificial diet and a secondary prey. J. Econ. Entomol..

[36] Cantón P.E., Bonning B.C. (2020). Extraoral digestion: outsourcing the role of the hemipteran midgut. Curr. Opin. Insect. Sci..

[37] Wu S., Deng W., Li M. (2020). Analysis of chemosensory genes in full and hungry adults of *Arma chinensis* (Pentatomidae) through antennal transcriptome. Front. Physiol..

[38] Shi Y.Y., Yan W.Y., Huang Z.Y., Wang Z.L., Wu X.B., Zeng Z.J. (2013). Genomewide analysis indicates that queen larvae have lower methylation levels in the honey bee (*Apis mellifera*). Naturwissenschaften.

[39] Livak K.J., Schmittgen T.D. (2001). Analysis of relative gene expression data using real-time quantitative PCR and the 2^-ΔΔCT^ method. Methods.

[40] Trott O., Olson A.J. (2010). AutoDock Vina: improving the speed and accuracy of docking with a new scoring function, efficient optimization, and multithreading. J. Comput. Chem..

[41] Xiao H., Ye X., Xu H. (2020). The genetic adaptations of fall armyworm *Spodoptera frugiperda* facilitated its rapid global dispersal and invasion. Mol. Ecol. Resour.

[42] Meslin C., Mainet P., Montagné N. (2022). *Spodoptera littoralis* genome mining brings insights on the dynamic of expansion of gustatory receptors in polyphagous Noctuidae. G3 (Bethesda).

[43] Sun Y.L., Jiang P.S., Dong B.X., Tian C.H., Dong J.F. (2022). Candidate chemosensory receptors in the antennae and maxillae of Spodoptera frugiperda (J.E. Smith) larvae. Front. Physiol..

[44] Weirauch C., Schuh R.T., Cassis G. (2019). Revisiting habitat and lifestyle transitions in Heteroptera (Insecta: Hemiptera): insights from a combined morphological and molecular phylogeny. Cladistics.

[45] Wu G., Wu C., Dewer Y. (2023). Comparative genomics reveals evolutionary drivers of the dietary shift in Hemiptera. Bull. Entomol. Res..

[46] Robertson H.M. (2019). Molecular evolution of the major arthropod chemoreceptor gene families. Annu. Rev. Entomol..

[47] Zhang J., Liang Q., Xia Y. (2021). Behavioral response of the tropical bed bug, *Cimex hemipterus* (Hemiptera: cimicidae) to carbon dioxide. J. Econ. Entomol..

[48] Wanner K.W., Robertson H.M. (2008). The gustatory receptor family in the silkworm moth *Bombyx mori* is characterized by a large expansion of a single lineage of putative bitter receptors. Insect Mol. Biol..

[49] Miyamoto T., Amrein H. (2014). Diverse roles for the *Drosophila fructose* sensor Gr43a. Fly.

[50] Liu X.L., Sun S.J., Hou W., Zhang J., Yan Q., Dong S.L. (2020). Functional characterization of two spliced variants of fructose gustatory receptor in the diamondback moth, *Plutella xylostella*. Pestic. Biochem. Physiol..

[51] Yang L., Agramonte N., Linthicum K.J., Bloomquist J.R. (2021). A survey of chemoreceptive responses on different mosquito appendages. J. Med. Entomol..

[52] Qin Z., Wu J., Qiu B., Ali S., Cuthbertson A.G.S. (2019). The impact of *Cryptolaemus montrouzieri* mulsant (Coleoptera: coccinellidae) on control of *Dysmicoccus neobrevipes* beardsley (Hemiptera: pseudococcidae). Insects.

